# Metagenomic next-generation sequencing and proteomics analysis in pediatric viral encephalitis and meningitis

**DOI:** 10.3389/fcimb.2023.1104858

**Published:** 2023-04-21

**Authors:** Yi-Long Wang, Xiao-Tong Guo, Meng-Ying Zhu, Yu-Chen Mao, Xue-Bin Xu, Yi Hua, Lu Xu, Li-Hua Jiang, Cong-Ying Zhao, Xin Zhang, Guo-Xia Sheng, Pei-Fang Jiang, Zhe-Feng Yuan, Feng Gao

**Affiliations:** ^1^ Department of Neurology, Children’s Hospital, Zhejiang University School of Medicine, Hangzhou, Zhejiang, China; ^2^ Children’s Hospital, Zhejiang University School of Medicine, National Clinical Research Center For Child Health, Hangzhou, Zhejiang, China

**Keywords:** viral encephalitis (VE), viral meningitis (VM), human enterovirus (HEV), metagenomic next-generation sequencing (mNGS), MS-based proteomics, diagnostic biomarkers

## Abstract

**Introduction:**

Early and accurate identification of pathogens is essential for improved outcomes in patients with viral encephalitis (VE) and/or viral meningitis (VM).

**Methods:**

In our research, Metagenomic next-generation sequencing (mNGS) which can identify viral pathogens unbiasedly was performed on RNA and DNA to identify potential pathogens in cerebrospinal fluid (CSF) samples from 50 pediatric patients with suspected VEs and/or VMs. Then we performed proteomics analysis on the 14 HEV-positive CSF samples and another 12 CSF samples from health controls (HCs). A supervised partial least squaresdiscriminant analysis (PLS-DA) and orthogonal PLS-DA (O-PLS-DA) model was performed using proteomics data.

**Results:**

Ten viruses in 48% patients were identified and the most common pathogen was human enterovirus (HEV) Echo18. 11 proteins overlapping between the top 20 DEPs in terms of P value and FC and the top 20 proteins in PLS-DA VIP lists were acquired.

**Discussion:**

Our result showed mNGS has certain advantages on pathogens identification in VE and VM and our research established a foundation to identify diagnosis biomarker candidates of HEV-positive meningitis based on MS-based proteomics analysis, which could also contribute toward investigating the HEV-specific host response patterns.

## Background

Viruses are the most prevalent cause of central nervous system (CNS) infections in pediatric patients; encephalitis and meningitis caused by viruses are called viral encephalitis (VE) and viral meningitis (VM), respectively (Hasbun et al., 2019; Li et al., 2022). The incidence rate of VE and/or VM is 10–20 cases/100,000 children/year globally (Hviid and Melbye, 2007; Michos et al., 2007). Although VE and/or VM are considered to be self-limiting, severe and even death cases of uncontrolled inflammation could occur. Thus, a viral infection of the CNS is potentially life-threatening in pediatric patients because of the associated mortality and morbidity (Okike et al., 2018; Ramchandar et al., 2021). To diagnose CNS infections, traditional laboratory methods, including culture, serological testing, and polymerase chain reaction (PCR) assay, rely on a series of tests. Moreover, the culture method for viable microorganisms takes at least 48 h to assess virulent colonies. Furthermore, pathogens of ~50% of patients with CNS infection remain unidentified (Ai et al., 2017; Anh et al., 2021). Therefore, a rapid and sensitive pathogen identification approach is essential to improve therapeutic measures and outcomes in patients with VE and/or VM. The primary pathogens responsible for VE and VM are associated with regional differences, seasonal variations, and detection methodology (Anh et al., 2021). Metagenomic next-generation sequencing (mNGS) can identify a broad range of pathogens, including bacteria, viruses (Miller et al., 2019; Wilson et al., 2019; Xing et al., 2020), parasites (Wang et al., 2015), and fungi (Christopeit et al., 2016) by means of a single test, and it is a promising approach for the diagnosis of infectious diseases (Goldberg et al., 2015; Forbes et al., 2017). Nonetheless, the low relative abundance of the virus genome compared with high noise (mainly reads from host-derived material) in clinical specimens presents a challenge to viral mNGS (Bukowska-Ośko et al., 2016). Recently, significant methodological improvements in mNGS assay have been achieved to help save time and cost (Manso et al., 2017; Schlaberg et al., 2017; Manso et al., 2020).

CSF contains proteins, enzymes, and other substances that can help detect infections in the CNS (Sathe et al., 2018). The molecular mechanism of a viral infection is dependent on the structure and biology of the virus. The variability of the virus and the complex virus-host interaction make studying viral infections difficult, and a novel and accurate technique is urgently needed (Milewska et al., 2020). MS, using a peptide-level amino acid sequence, provides unbiased identification and quantification of proteins in human samples (Domon and Aebersold, 2006). It is proving valuable in identifying pathogen type–specific CSF protein profiles and for providing insights into specific virulence mechanisms. MS-based proteomics could be important diagnostic biomarkers for infectious CNS diseases (Bakochi et al., 2021).

mNGS has been widely studied in infectious diseases of the central nervous system, but few in proteomic analysis (Piantadosi et al., 2021; Zhu et al., 2022; Li et al., 2023; Lin et al., 2023). In our research, we collected 50 CSF samples from children with suspected VE and/or VM in Zhejiang Province in 2021. Pathogens were identified by using mNGS from both RNA and DNA, then mass spectrometry (MS)-based proteomics and data-independent acquisition (DIA) analyses were used to generate CSF proteome maps of HEV-positive group. Our study aimed at examining the utility of cerebrospinal fluid mNGS in identifying potential viral etiologies in suspected VE and/or VM patients and finding useful diagnosis biomarker candidates for HEV-positive meningitis based on the results of the MS-based proteomics analysis which may also contribute toward investigating the HEV-specific host response patterns.

## Methods

### Patients and CSF sample collection

We selected 50 pediatric patients with suspected VEs and/or VMs from the Children’s Hospital of Zhejiang University School of Medicine, who were hospitalized between January 1, 2021, and November 30, 2021. We performed mNGS on the CSF samples of the selected patients. Written informed consent was obtained from the children’s guardians. Subsequently, we performed proteomics analysis of HEV-positive CSF samples and CSF samples of HCs. The inclusion and exclusion criteria for performing mNGS are provided in the **Supplementary Material** (Kneen et al., 2012; Venkatesan et al., 2013). To collect CSF, we performed a lumbar puncture; the CSF specimens were sent for routine laboratory biochemical analysis and subjected to mNGS within 24 h. The remaining (0.3–0.8 mL) of the CSF sample was centrifuged at 4°C for 10 min at 1,000 g and stored at −80°C until assayed.

### DNA and RNA extraction, library preparation, and sequencing

All samples were extracted with QIAamp^®^ UCP Pathogen DNA Kit (Qiagen). Tween 20 and Benzonase (Qiagen) are used to remove DNA from human samples according to the manufacturer’s instructions (Amar et al., 2021). A QIAamp^®^ Viral RNA Kit was used to extract RNA, and a Ribo-Zero rRNA Removal Kit (Illumina) was used to remove ribosomal RNA. cDNA was synthesized with reverse transcriptase and dNTP (Thermo Fisher). The construction of the selected DNA and cDNA library was done using Nextera XT DNA Library Prep Kit (Illumina, San Diego, CA) (Miller et al., 2019). Quality control for the library was performed using a Qubit dsDNA HS Assay Kit and a High Sensitivity DNA kit (Agilent) on an Agilent Bioanalyzer 2100. Then, the library pools were loaded onto an Illumina NextSeq CN500 sequencer for 75 cycles of single-end sequencing of 20 million reads for each library. Negative control (NC) is a peripheral blood mononuclear cell sample of 105 cells/mL prepared alongside each batch with the same protocol (Amar et al., 2021), and sterile deionized water was used as a nontemplate control (NTC) alongside the specimens (Li et al., 2018).

### Bioinformatics analyses of mNGS

We used Trimmomatic to eliminate adapter contamination, poor-quality reads, duplicated reads, and those <50 bp. Around 20 million read pairs were produced per sample. K-complexity with default parameters was used to remove low-complexity reads. Human DNA sequence was distinguished and excluded using Burrows-Wheeler Aligner software, and human gene hg38 was used as a reference. Kraken2 v2.0.8 beta was used to build reference databases, and the taxonomy of sequences was achieved using Kraken2 v2.0.8 beta. The number of mapped reads was extracted from Bracken using default parameters. When mapping against a reference genome, the presence of a virus was deemed positive if three or more nonoverlapping sequences from the genome were identified. A read per million ratio (RPM-r) of ≥10 is defined as a positive detection. The RPM-r is named RPM sample/RPMNC (i.e., the RPM of a certain species or genus in CSF samples divided by the RPM of the NC). In addition, to minimize cross-species amplification among closely related microorganisms, we considerably reduced 5% and 10% RPM of species and genus, respectively, when microbes appeared in NTC to minimize misalignment of cross-species and false positive rate. All aligned reads were assembled by MEGAHIT using default parameters. Resulting contigs were aligned to HEV reference genome (E9: GCA_008766535.1; E18: GCA_008800315.1; E30: GCA_008800595.1; Coxsakie B3: GCF_002816685.1) using BLASTn with an e-value of 1e-10 and processed using homemade Perl script.

### CSF Peptide preparation, liquid chromatography–mass spectrometry analysis, and identification and quantitation of protein

Extraction, quantification, and digestion of CSF proteins were performed before liquid LC-MS analysis according to standard protocols. LC-MS/MS was performed on targeted proteins in CSF and serum samples for data-dependent acquisition (DDA) mode and DIA mode according to the method used in our previous study (Wang et al., 2022). Using Proteome Discoverer 2.2 (PD 2.2; Thermo Fisher Scientific), we searched the MS/MS spectra separately against homo_sapiens_uniprot_2021_7_15. Precursor mass tolerance was set to 10 ppm 0.02 Da for both product ions and precursors. Methionine (M) oxidation was set as a dynamic modification, whereas carbamidomethyl (C) was fixed. N-terminal acetylation was set as a modification in PD 2.2. The cleavage specificity was trypsin, with a maximum of two missed cleavage peptides allowed. The credibility of peptide–spectrum matches (PSMs) was set to >99% for PSM identifications, and identifying proteins required at least one unique peptide. We set the false discovery rate (FDR) threshold at 1.0% for identifying PSMs and proteins. According to the peptide and ion pair selection criteria, the qualified peptides and product ions were chosen from the resulting spectra to create a target list. In accordance with the target list, DIA data were imported to obtain the ion-pair chromatographic peaks. To determine the qualitative and quantitative properties of peptides, peak areas and ions were matched. The protein quantification results were analyzed by performing a *t*-test. The Q value cut-off for the precursor ion was set at 0.01.

### Data analysis

In order to compare the protein content of each sample, principal component analysis (PCA) was used as a pre-processing dimension reduction method. A value of *p <*0.05 and fold change (FC) ≥ 1.5 or FC ≤ 0.67 were considered significant for differentially expressed proteins (DEPs) between experimental and control groups. The DEPs were visualized with a heatmap produced using the R packages pheatmap and ggplot2. The potential of a specific protein biomarker was then determined using supervised partial least squares-discriminant analysis (PLS-DA) and orthogonal PLS-DA (O-PLS-DA) using R scripts (Ropls package). Using the variable importance projection (VIP) score, the relative magnitude of the changes observed between the experimental and control groups was assessed and proteins with VIP values ≥ 1 were considered the best classifiers. Candidate biomarkers were defined as proteins overlapping between the top 20 DEPs (defined as FC >1.5 or or ≤ 0.67 and P < 0.05) and the top 20 proteins in the O-PLS-DA VIP list. All values are presented as means ± SEM. Student *t*-test was performed using Prism 8.4.3 software. A value of *p <*0.05 is considered statistically significant.

### Functional analysis of protein and DEPs

Gene Ontology (GO) and InterPro (IPR) analyses were performed to characterize DEPs against the nonredundant protein database (Pfam, SMART, ProDom, ProSite, and PANTHER). Kyoto Encyclopedia Genes and Genomes (KEGG) and Clusters of Orthologous Groups (COG) analyses were performed to predict the possible pathway and family.

## Results

### Clinical characteristics of participants

Clinical characteristics and outcomes of the hospitalized participants are summarized in **Table 1**. The median age of this group was 4.6 years (interquartile range [IQR], 2.4–7.5 years) with 27 men and 23 women, and the median length of hospital stay was 11 days (IQR, 7-15 days), 86% of the patients had fever, 52% had headaches, 50% had vomiting, and 68% of the patients had altered mental status. Regarding the CSF parameters, the median time from onset to CSF collection was 4 days (IQR, 3-7 days), the median WBC level was with 35 cells/μL (IQR, 7.5-89.8 cells/μL), the median level of protein was 268.7 mg/L (IQR, 204.1–340.6 mg/L), and the median glucose level was 3.5 mmol/L (IQR, 3.3-3.8 mmol/L). All patients underwent a cranial magnetic resonance imaging (MRI) examination, 38% of them were abnormal. Of 50 children, 5 have their spinal checked by MRI, and 3 of them were abnormal and all were diagnosed with autoimmune encephalitis. Twenty-three out of 50 children underwent the conventional electroencephalogram (EEG) examination and 10 (43%) of them were abnormal. Forty-three patients underwent visual electroencephalogram (VEEG), and 18 (42%) of them were abnormal. In terms of the therapy situation, almost all patients were treated with Acyclovir except one. Of the children, 40% received glucocorticoid therapy, 22% used intravenous immunoglobulin. Regarding the prognosis, 80% of the patients made full recovery when leaving the hospital, while 20% got partial improvement, 12% left the sequela and 8% were treated the ICU.

**Table 1:** Demographics and clinical statistics of research subjects (n = 50).

Male sex, n (%)	27 (54%)
Age (years) mean (range)	5.6 (0.3–13.7)
Length of stay, median days (IQR)	11 (7–15)
Syndrome	
Viral encephalitis, n (%)	23 (46%)
Viral meningitis	19 (38%)
Viral meningoencephalitis	2 (4%)
Autoimmune encephalitis	4 (8%)
Acute cerebellitis	2 (4%)
Presenting symptoms	
Fever, n (%)	43 (86%)
Vomiting, n (%)	25 (50%)
Seizures, n (%)	20 (40%)
Headache, n (%)	26 (52%)
Altered mental status, n (%)	34 (68%)
Time from onset to CSF collection, median days(IQR)	4 (3–7)
CSF Parameters	
Nucleated cells cells/µL, median (IQR)	35 (7.5–89.8)
Proteins mg/dL, median (IQR)	268.7 (204.1–340.6)
Glucose mg/dL, median (IQR)	3.5 (3.3–3.8)
Abnormality of examinations	
Cranial MRI, (%)	38
EEG, (%)	43
VEEG, (%)	42
Therapy	
Acyclovir, n (%)	49 (98%)
Glucocorticoid, n (%)	20 (40%)
Intravenous immunoglobulin	11 (22%)
Prognosis	
Full recovery, n (%)	40 (80%)
Partial recovery, n (%)	3 (6%)
Sequelae, n (%)	7 (14%)
Admitted to ICU, n (%)	4 (8%)
Total patients	50

CSF, cerebrospinal fluid; IQR, interquartile range; MRI, magnetic resonance imaging; EEG, electroencephalography; VEEG, video electroencephalography.

Altered mental status: manifesting as reduced consciousness or altered cognition, personality, or behavior.

### mNGS analysis of CSF samples

**Figure 1:**
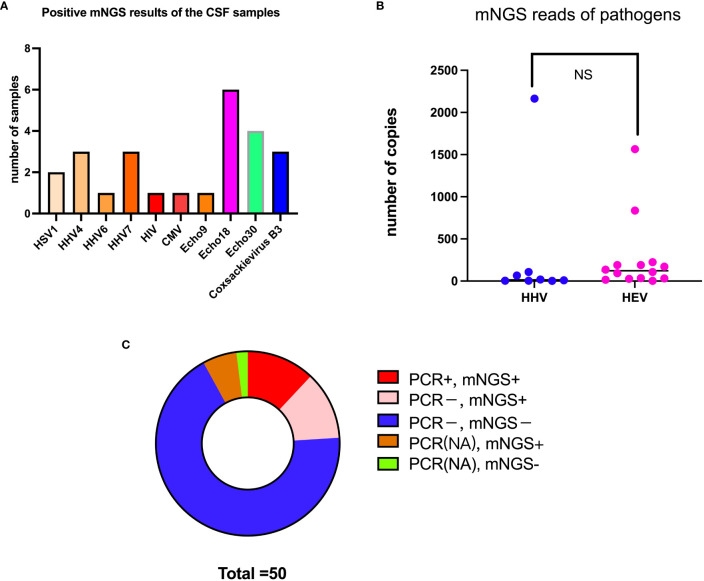
mNGS analysis results of the CSF samples. **(A)** Total number (besides the bars) of samples with a positive result for each pathogen (x-axis). **(B)** Comparison of the number of reads between HEV and HHV. **(C)** Comparison of detection rate between mNGS and PCR analysis. NS, no significant differences.

mNGS was applied to 50 CSF samples. As shown in **Table 2** and **Figure 1A**, 10 virus types were identified in 23 of 50 (48%) patient CSF samples: Echo18, the leading pathogen, was detected in 26.09% of CSF samples tested, followed by Echo30 (17.39%), CB3 (13.04%), human herpes virus (HHV) 7 (13.04%), HHV-4 (13.04%), herpes simplex virus 1 (HSV-1; 8.70%), HHV-6 (4.35%), Echo 9 (4.35%), human immunodeficiency virus (HIV; 4.35%) and cytomegalovirus (CMV; 4.35%). Echo18, Echo30, Coxsackie B3, and Echo 9 all belong to the HEV species. As shown in **Figure 1B**, the mean number of reads for HEV was 258.9 per sample (range, 2–1565); the number of reads for HEV was similar to that of other viruses (*p* > 0.05), and there was no significant difference in the number of reads between Echo 30 and Echo 18 (*p* > 0.05). The combined analysis of mNGS assay results and clinical diagnosis showed that 86% of HEV-positive participants (n = 14) were diagnosed with VM, whereas 78% of HHV-positive participants (n = 9) were diagnosed as VE. Simultaneously, 46 of the 50 CSF samples were sent to clinical laboratories for routine HEV PCR analysis. The results of HEV-specific PCR testing showed that 6 of the 46 CSF samples were HEV-positive, whereas mNGS assay results showed that 14 of the 46 CSF samples were HEV-positive, indicating a significant improvement in HEV detection rate with mNGS (**Figure 1C**).

**Table 2:** Presentation of subjects for which CSF testing was positive by any method.

Subject	CSF culture	HSV 1 PCR	HSV 2 PCR	EV PCR	CSF mNGS	Number of reads	Diagnosis
2	no organisms	negative	not done	positive	Echo18	93	VM
3	no organisms	ND	negative	positive	Coxsackie B3	224	VM
4	no organisms	negative	negative	negative	Echo30	171	VM
11	no organisms	negative	negative	negative	Echo30	2	VM
13	no organisms	ND	negative	negative	HHV7	18	VE
14	no organisms	ND	ND	ND	EBV	9	VE
					HIV	530	
16	no organisms	negative	negative	positive	Echo18	1565	VM
17	no organisms	ND	negative	negative	HHV7	4	VE
18	no organisms	negative	not done	positive	Echo18	191	VM
21	no organisms	ND	negative	negative	HSV1	65	VE
24	no organisms	negative	negative	ND	CoxsackieB3	32	VM
25	no organisms	negative	negative	negative	Echo18	15	VM
28	no organisms	ND	negative	negative	HHV6	108	VE
29	no organisms	negative	negative	positive	CoxsackieB3	136	VM
30	no organisms	ND	negative	negative	EBV	3	VE
34	no organisms	negative	negative	negative	Echo18	191	VM
35	no organisms	negative	ND	positive	Echo30	108	VE
36	no organisms	negative	negative	negative	Echo9	26	VM
37	no organisms	negative	ND	ND	Echo18	35	VME
40	no organisms	negative	negative	negative	EBV	4	VE
41	no organisms	negative	negative	negative	HHV7	4	VE
42	no organisms	negative	negative	ND	Echo30	836	VME
46	no organisms	positive	negative	negative	HSV1	2164	VE

CSF, cerebrospinal fluid; EV, enterovirus; HHV-6, human herpes virus 6; HHV-7, human herpes virus 7; EBV, Epstein–Barr virus; HSV, herpes simplex virus; mNGS, metagenomic next-generation sequencing; PCR, polymerase chain reaction; ND, not detected; VE, viral encephalitis; VM, viral meningitis; VME, viral meningoencephalitis.

### Proteomics analysis of patients with HEV-positive meningitis

**Figure 2:**
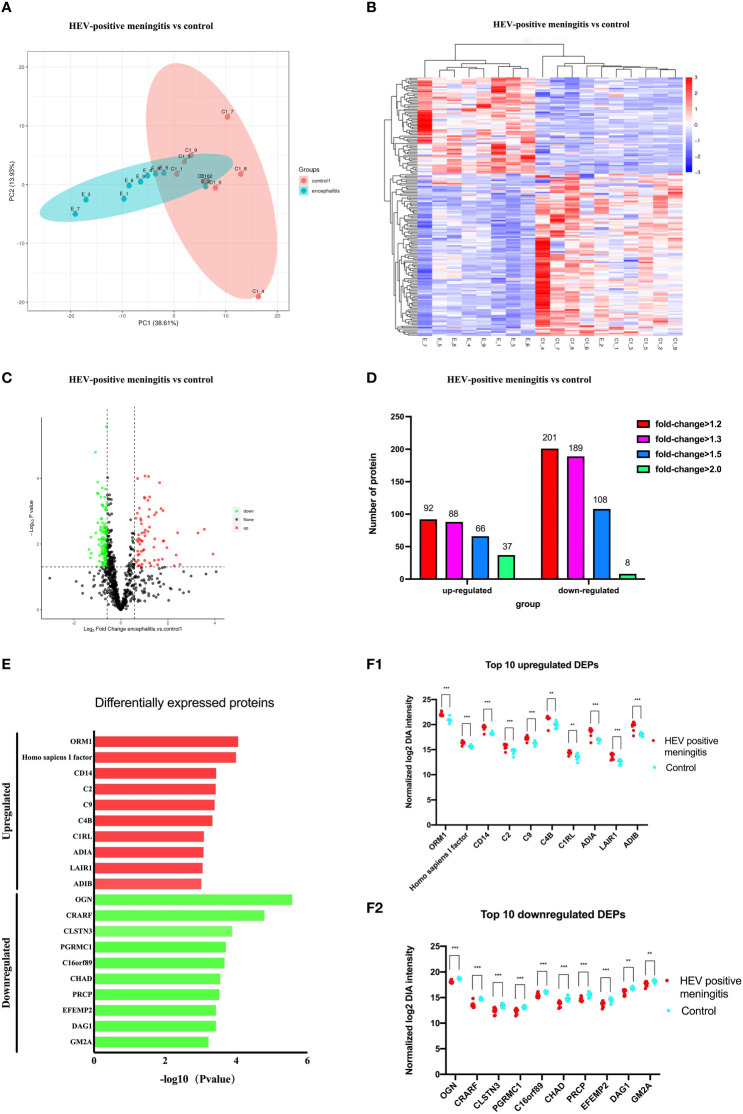
Comparison of protein expression profiles between patients with encephalitis and normal controls. **(A)** PCA of whole proteins from proteomics data. **(B)** Expression heatmaps of differentially expressed proteins. **(C)** Volcano plot of differentially expressed proteins between two groups: ∣log2FC∣ > 2, FDR < 0.01. **(D)** The up- and downregulated fold changes (log2) of proteins are colored according to each panel key. **(E)** Names of the top 10 up- (red) or downregulated (green) proteins between patients with HEV-positive meningitis and normal controls are listed in a bar chart. **(F)** Comparison of top 10 up- (B1) or downregulated (B2) protein abundance is shown by dot plots between patients with EV-positive encephalitis (red) and normal controls (blue). Relative expression levels (2^−ΔΔCt^) of these DEPs are shown according to the log scale. Corresponding protein names are listed at the bottom of the chart. *p<0.05, **p<0.01, ***p<0.001.

**Figure 3:**
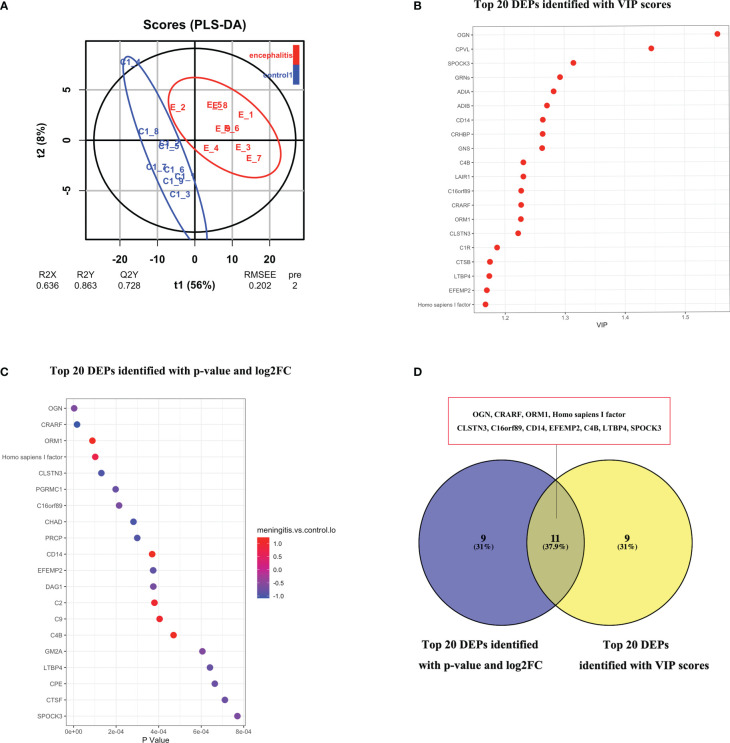
**(A)** O-PLS-DA analysis of DEPs between children with with HEV-positive meningitis and normal controls. **(B)** Top 20 DEPs identified with most importance using O-PLS-DA. **(C)** Top 20 DEPs identified with P value and FC. **(D)** 11 DEPs identified with top-ranked P value, FC and VIP scores.

The mNGS assay results revealed that HEV was the primary causative pathogen of VE and/or VM in participants. To better understand the specific virulence mechanisms of HEV in pathogen type–specific host response patterns, we performed proteomics analysis on the 14 remaining HEV-positive CSF samples and another 12 CSF samples from HCs. A PCA analysis was performed on CSF proteomics data to visualize the overall trend in protein expression levels. It demonstrated that HEV-positive meningitis patients and HCs expressed proteins differently (**Figure 2A**). Subsequently, we identified 816 proteins, including 66 upregulated proteins and 108 downregulated proteins, in the CSF samples of the patients in the HEV-positive group, compared with HCs by analysis with DIA mode (**Figures 2B–D**). The top 10 upregulated or downregulated proteins are illustrated in a bar chart (**Figures 2E, F**). The top 20 DEPs with the top-ranked P value and FC are demonstrated in **Figure 3C**.

**Figure 4:**
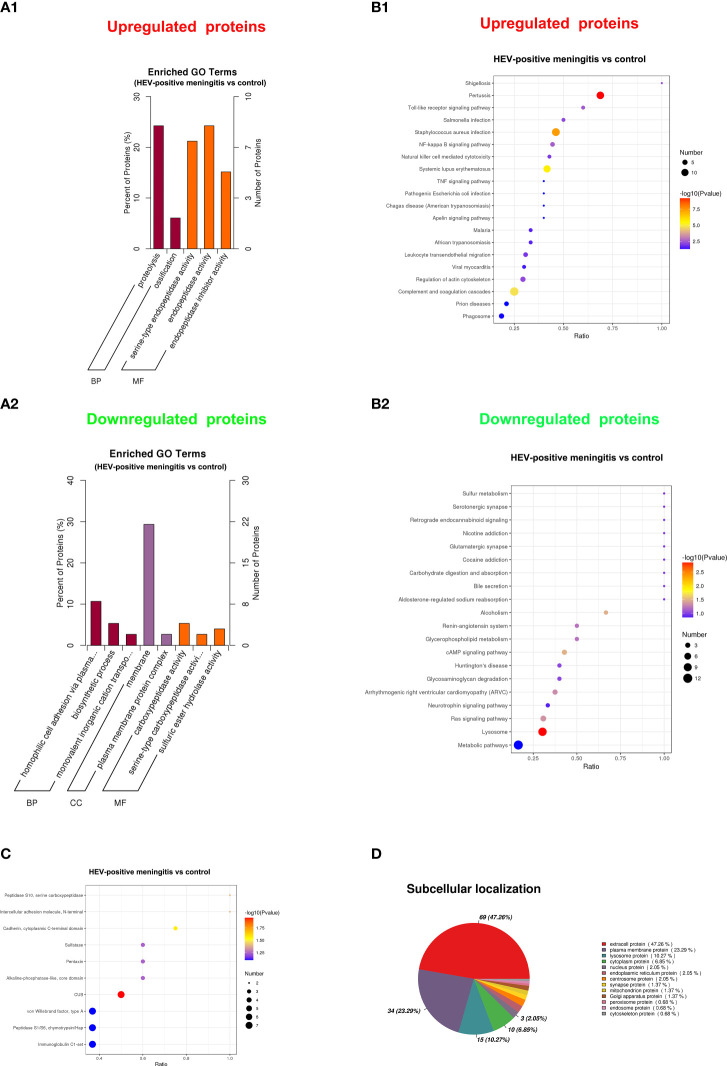
Functional analysis of DEPs between patients with encephalitis and normal controls. **(A)** GO enrichment bar chart of DEPs presents the number and percentage of DEPs enriched in a biological process (BP), cellular component (CC), and molecular function (MF) between the patients with encephalitis and normal controls. **(A1)** upregulated expressed proteins; **(A2)** downregulated expressed proteins. **(B)** The bubble chart shows the KEGG pathway enrichment analysis of DEPs between two groups. **(B1)** upregulated expressed proteins; **(B2)** downregulated expressed proteins. **(C)** InterPro (IPR) annotation of DEPs between patients with encephalitis and normal controls and the top 10 domain annotations represented in the chart. **(D)** Subcellular localization of DEPs between patients with encephalitis and normal controls.

To identify the potential functions of DEPs, we conducted GO, KEGG, IPR, and subcellular localization analyses. The GO enrichment analysis revealed that the upregulated proteins were primarily associated with serine-type endopeptidase activity, endopeptidase activity, proteolysis, ossification, and endopeptidase inhibitor activity (**Figure 4A1**). Alternatively, the downregulated proteins were primarily involved in carboxypeptidase activity, membrane, homophilic cell adhesion *via* plasma membrane adhesion molecules, serine-type carboxypeptidase activity, and plasma membrane protein complex (**Figure 4A2**). According to the KEGG pathway analysis results, the upregulated proteins are principally involved in infections such as pertussis, *Staphylococcus aureus* infection, and systemic lupus erythematosus, as well as in complement and coagulation cascades, NF-kappa B signaling pathway, and toll-like receptor signaling pathway (**Figure 4B1**), whereas the downregulated proteins are primarily enriched in the lysosome, cAMP signaling pathway, and Ras signaling pathway, as well as in conditions such as alcoholism and arrhythmogenic right ventricular cardiomyopathy (**Figure 4B2**). In addition, results of the IPR annotation analysis showed that DEPs were primarily enriched in CUB, C-cadherin cytoplasmic domain, the peptidase S10 family of serine carboxypeptidase, and the N-terminal domain of intercellular adhesion molecule (**Figure 4C**). Subcellular localization assay results revealed that DEPs are mainly related to extracellular protein (47.26%), plasma membrane protein (23.29%), and lysosome protein (10.27%) (**Figure 4D**).

In order to reduce data dimensionality, PLS-DA and O-PLS-DA were implemented in consideration of model fit and predictability. As showed in **Figure 3A**, the total variation between MOG patients and HCs divided into two components (R2 X = 0.636, R2 Y = 0.863, Q2 Y = 0.728), Demonstrating the value of CSF proteome change in identifying HEV-positive meningitis patients from healthy controls. In the O-PLS-DA model, the corresponding VIP score was calculated to measure protein identification performance. There were 82 differentially abundant proteins identified with VIP scores >1 and the top 20 proteins with highest VIP scores in O-PLS-DA were listed in **Figure 3B**. 11 proteins including Mimecan (OGN), Complement-activating component of Ra-reactive factor (CRARF), Alpha-1-acid glycoprotein 1 (ORM1), Homo sapiens I factor, Calsyntenin-3 (CLSN3), UPF0764 protein C16orf89 (C16orf89), Monocyte differentiation antigen CD14 (CD14), Extracellular matrix protein 2 (EFEMP2), Complement C4-B (C4B), Latent-transforming growth factor beta-binding protein 4 (LTBP4), Sparc/osteonectin, cwcv and kazal-like domains proteoglycan (Testican) 3 (SPOCK3) overlapping between the top 20 DEPs (defined as FC >1.5 or ≤ 0.67 and P < 0.05) and the top 20 proteins in the O-PLS-DA VIP list were defined as candidate proteins (**Figure 3D**).

## Discussion

Viruses are the leading causative pathogens in pediatric CNS infections. Viral infection of the CNS in pediatric patients is often associated with high mortality rates and long-term neurological consequences. The pathogenic virus types of pediatric VE and/or VM are geographically dependent: in Sweden, HEV, HSV-2, varicella zoster virus (VZV), and HHV-6 are the primary pathogens of CNS infections (Lindström et al., 2021); in France, HSV and VZV are the most frequent causative pathogens in patients with acute encephalitis (Le Maréchal et al., 2021); and in Vietnam, HEV is the most common virus followed by hepatitis B virus (Anh et al., 2021). However, many patients with suspected viral infection of the CNS do not present with clear infectious etiologies. The mNGS is a promising tool for identifying such pathogens. In our research, mNGS was used to study the epidemiology of VE and/or VM in Zhejiang Province in 2021. Similar to the results of previous studies (Chen et al., 2018; Sun et al., 2019), our data showed that HEV remains the primary pathogen of pediatric VE and/or VM based on the evidence that 28% (14 of 50) of CSF samples of all enrolled patients in Zhejiang Province in China were HEV-positive. Moreover, Echo 18 replaced Echo 30 as the most frequent serotype. In our research, the HEV-specific PCR test results showed that six of the 46 CSF samples were positive, whereas 14 of the 46 CSF samples were positive according to the mNGS detection analysis, indicating a significant improvement in the detection rate when using mNGS compared with traditional PCR analysis, a conclusion consistent with that of previously published reports (Wilson et al., 2019; Anh et al., 2021).

Three patients, among the 14 patients whose CSF samples were HEV-positive, presented with abnormal white matter lesions in their cranial MRI report. This outcome is consonant with that of previous work (Chen et al., 2020). The MRI reports of two HSV1-positive patients suggested abnormal signal intensity in the temporal and/or frontal lobes, a result in accordance with the published guidelines (Tunkel et al., 2008). Two of four HHV-7-positive patients were admitted to the ICU, indicating a poor prognosis of HHV-7 infection (Schwartz et al., 2014). Despite the encouraging results obtained from both previous work and this study on mNGS usefulness in identifying pathogens in infectious diseases, mNGS is also considerably expensive for many families. This cost may be mitigated by covering mNGS costs in health insurance plans.

Based on the proteomic analysis of the CSF samples obtained from children with HEV-positive meningitis, we found that a large number of upregulated proteins in the CSF, including C2, C9, C4B, C1RL, ADIA, LAIR1, ADIB, C1R, VCAM1, ADA2, SERPINA1, C5, CD14, SPP1, epididymal secretory sperm binding protein, ICAM2, and IGHV2–26, were related to complement and coagulation cascades, NF-kappa B signaling pathway, and toll-like receptor signaling pathway. C9 ADIA, LAIR1, and ADIB, all of which are associated with complement and coagulation cascades, were profoundly overexpressed in the CSF samples of patients with HEV-positive meningitis. Inflammatory glycoproteins chitinase-3-like protein 1, and chitinase-3-like protein 2 (YKL-39) have emerged as biomarkers for multiple sclerosis (MS), demyelinating diseases, VM, and acute bacterial meningitis. In our study, CSF samples from patients with HEV-positive meningitis showed obviously elevated expression levels of YKL-40 and YKL-39. The expression level of GM2A, CTSF, ARSA, GNS, GAA, CTSD, LAMP2, BDNF, GRIA4, and ATP1B1, all of which are relevant to lysosome and cAMP signaling pathway, was downregulated in HEV-positive CSF samples.

In order to reduce data dimensionality, PLS-DA and O-PLS-DA were implemented to access model fit and predictability. 11 proteins including Mimecan (OGN), Complement-activating component of Ra-reactive factor (CRARF), Alpha-1-acid glycoprotein 1 (ORM1), Homo sapiens I factor, Calsyntenin-3 (CLSN3), UPF0764 protein C16orf89 (C16orf89), Monocyte differentiation antigen CD14 (CD14), Extracellular matrix protein 2 (EFEMP2), Complement C4-B (C4B), Latent-transforming growth factor beta-binding protein 4 (LTBP4), Sparc/osteonectin, cwcv and kazal-like domains proteoglycan (Testican) 3 (SPOCK3) overlapping between the top 20 DEPs and the top 20 proteins in the O-PLS-DA VIP list were identified. Mimecan, Complement-activating component of Ra-reactive factor and Alpha-1-acid glycoprotein 1 acts as the most important variable to differentiate HEV-positive meningitis patients from HCs. Mimecan, also known as osteoglycin (OGN), is a class III small leucine-rich proteoglycans (SLRPs) member (Iozzo, 1997). OGN has been shown to be an NFκB/IKK-dependent gene related to the immunity in mice embryonic fibroblasts (Zandi et al., 1997). In another lipopolysaccharide (LPS)-treated rat model, OGN expression was increased in the regulation of Toll-like receptor (TLR) 4 pathway (Haglund et al., 2008). Thus, we can assume that differentially expressed OGN between HEV-positive group and HCs participates in inflammatory immune responses and has potential as a therapeutic target candidate. Complement-activating component of Ra-reactive factor (CRARF), a C-dependent bactericidal factor binding specifically to Ra chemotype Salmonella strains, is present in a wide variety of vertebrates. CRARF was mapped to human 3q27-q28 and demonstrated to be essential in resisting infections by enteric microorganisms (Kawakami et al., 1984). In a hemolysis model system of LPS-coated E (ELPS), complement cascade was found to be activated by CRARF through the activation of C2 and C4 (Ji et al., 1988). Interestingly, our study also found C4B expression increased in CSF from HEV-positive group. Alpha-1 acid glycoprotein (AGP) is a serum protein expressed by the *orosomucoid* genes (ORM1 and ORM2) in the acute phase of inflammation responses (Treuheit et al., 1992; Fournier et al., 2000). The liver is the primary organ that produces AGP, which is also secreted by monocytes and alveolar macrophages (Fournier et al., 1999). Previous studies show that AGP1 is involved in the cellular initiation of inflammation and coagulation, and its plasma concentration could increase two to five fold during inflammation (Martyn et al., 1984; Sumanth et al., 2019). AGP in serum is a potential candidate biomarker for the diagnosis of HEV-71 infection (Wang et al., 2015). In our study, we found that the two subtypes of AGP were significantly elevated in the CSF sample obtained from patients with HEV-positive meningitis; this outcome can be a potential candidate biomarker panel for diagnosis of HEV-positive meningitis.

In general, our study provided etiological and epidemiological features of children diagnosed with acute VM and/or VE in Zhejiang Province in 2021 and showed that mNGS has certain advantages over traditional PCR assay to identify pathogens that cause VE and/or VM. We also established a foundation to identify diagnosis biomarker candidates of HEV-positive meningitis based on the results of the MS-based proteomics analysis, which could also contribute toward investigating the HEV-specific host response patterns.

Our research still has several limitations. This was a single-center study, the study cohort was relatively small, the investigation period was relatively short. Further validation of the potential protein biomarkers was impeded by insufficient CSF sample capacity. In terms of the guidelines, special immunoglobulin M (IgM) antibody tests in CSF samples of patients with encephalitis are helpful in diagnosis, but we did not conduct routine special IgM antibody tests of CSF samples, leading to the possible omission of pathogen diagnosis because of the rapid degeneration of pathogen nucleic acid before sample collection or the availability of very few viral sequences to be detected (Tunkel et al., 2008). Furthermore, several CSF samples were not sent for HEV-specific PCR tests as the physician’s judgment, which may influence the comparison results between PCR and mNGS. We will focus on and try to overcome these limitations in our next research.

## Conclusion

Overall, the present study yielded two interesting results. First, mNGS analysis revealed that HEV is the primary pathogen present in the CSF samples of children diagnosed with VE and/or VM in our hospital. Second, proteomics analysis could lead to the development of new biomarker candidates for HEV-positive VEs or VMs. This study might contribute toward improving the diagnosis of VE and/or VM and provide therapeutic targets for VE and/or VM.

## Data availability statement

The mass spectrometry proteomics data have been deposited to the ProteomeXchange Consortium (http://proteomecentral.proteomexchange.org) *via* the iProX partner repository with the dataset identifier PXD038291.

## Ethics statement

The studies involving human participants were reviewed and approved by the Ethics Committee of the Children’s Hospital of Zhejiang University School of Medicine (2019-IRB-054). Written informed consent to participate in this study was provided by the participants’ legal guardian/next of kin.

## Author contributions

FG and Y-LW conceived and designed this study, participated in data collection and analysis and reviewed and edited the manuscript. X-TG, M-YZ and X-BX were all involved in supervision, data collection, and critical review and revision of the whole manuscript. All authors contributed to the article and approved the submitted version.
