# Supplementary material for: Metagenomic next-generation sequencing and proteomics analysis in pediatric viral encephalitis and meningitis
Source: Front Cell Infect Microbiol. 2023 Apr 21;13:1104858. doi: 10.3389/fcimb.2023.1104858 (PMC10161730; doi:10.3389/fcimb.2023.1104858)
Supplement: Supplementary file 1 [file Presentation_1.pdf]

Supplementary Material

The inclusion criteria were as follows: (1) Meningitis was defined as the presence of symptoms consistent with meningitis and cerebrospinal fluid pleocytosis (CSF) (5> cells per L); (2) Encephalitis was defined according to the International Encephalitis Consortium as altered mental status (defined as decreased or altered level of consciousness, including change in personality, lethargy) for over 24 h with no alternative cause identified and two of the following: seizures, focal neurologic findings, electroencephalography (EEG) or magnetic resonance imaging (MRI) abnormalities suggestive of encephalitis, CSF pleocytosis and fever. The exclusion criteria were as follows: refusal to undergo lumbar puncture; any contraindication for such puncture; and a diagnosis of demyelinating, metabolic, toxic or neurological degenerative diseases.

The exclusion criteria were as follows: refusal to undergo lumbar puncture; any contraindication for such puncture; a diagnosis of demyelinating, metabolic, toxic, or neurological degenerative diseases; the etiology of CNS infection is bacterial and fungal.
